# Chondroma of the Nasal Septum

**DOI:** 10.7759/cureus.12941

**Published:** 2021-01-27

**Authors:** Munawar Peer, Jamie Tibbo, Ken Kao, Simon Kirby

**Affiliations:** 1 Medicine, Memorial University of Newfoundland, St. John's, CAN; 2 Otolaryngology - Head and Neck Surgery, Memorial University of Newfoundland, St. John's, CAN; 3 Biomedical Sciences, Memorial University of Newfoundland, St. John's, CAN; 4 Pathology, Memorial University of Newfoundland, St. John's, CAN

**Keywords:** chondroma, nasal septum, otolaryngology, pathology

## Abstract

Chondromas are benign cartilaginous tumours that rarely occur in the head and neck region. Only a limited number of cases have been reported involving the nasal septum. Here we report a case of a 55-year-old male that presented with a suspicious lesion involving his nasal septum and columella. The lesion was removed under general anesthestic using a combination of both columellar and hemitransfixion incisions. The lesion consisted of firm tan-white tissue measuring 2.5 cm. Histopathologic examination revealed a low-grade chondroid neoplasm with lobulated hyaline cartilage. No signs of ischemic change, significant pleomorphism, mitoses, or necrosis were present. This was consistent with the features of a chondroma rather than a low-grade chondrosarcoma. A chondroma should be considered in the differential diagnosis of nasal septum lesions. Surgical excision of the tumour is the preferred treatment option.

## Introduction

Chondromas are benign cartilaginous tumours that often occur in the small bones of hands and feet. The presence of cartilaginous tumours in the head and neck region are rare [1]. Amongst reported cases, the majority have occurred in the ethmoid sinus and maxilla [2]. We report a case of a chondroma of the nasal septum. Since 1842, only a limited number of nasal septum chondromas have been reported in the literature [3]. These patients often have symptoms including nasal obstruction, epistaxis, and pressure effects on surrounding structure [1,4]. Imaging and histopathological techniques have classically been used to differentiate a chondroma from malignant tumours such as a chondrosarcoma. This case was previously presented as a poster. (Poster: Peer MS, Tibbo J, Kao KR, Kirby SD. Chondroma of the Nasal Septum: A Case Report. Canadian Society of Otolaryngology-Head and Neck Surgery 73rd Annual Meeting; 2019).

## Case presentation

A 55-year-old male presented to the clinic with a suspicious growing lesion involving the nasal septum and columella. The lesion was firm and there was associated swelling of the distal aspect of the nasal septum. The nasal septum was severely deviated due to past untreated nasal fractures. A punch biopsy of the lesion was performed in the clinic under local anesthesia, which demonstrated fragments of necrotic cartilage and no malignancy. The lesion continued to grow in size, prompting excision under general anesthesia in the operating room. An inverted gull-wing incision was made and iris scissors were used to lift off the skin over the dorsum of the nose. The incision was then extended into a hemitransfixion incision into the nasal septum. Iris scissors were used to dissect around the lesion which encompassed bilateral lower cartilages. The excised lesion measured 2.5 x 1.5 x 1.1 cm and was sent for pathology.

**Figure 1:**
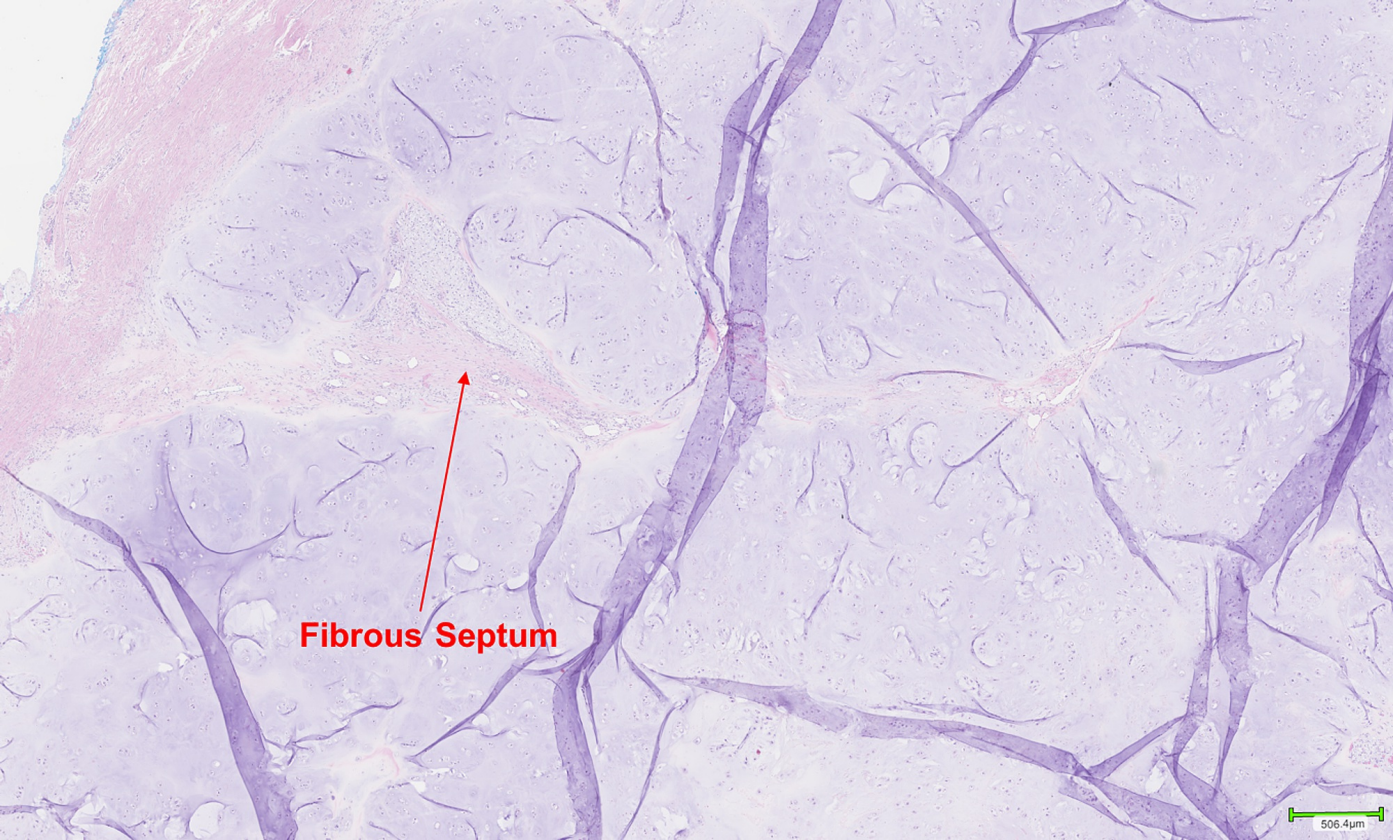
Low power image demonstrating lobulated pattern and fibrous septae.

**Figure 2:**
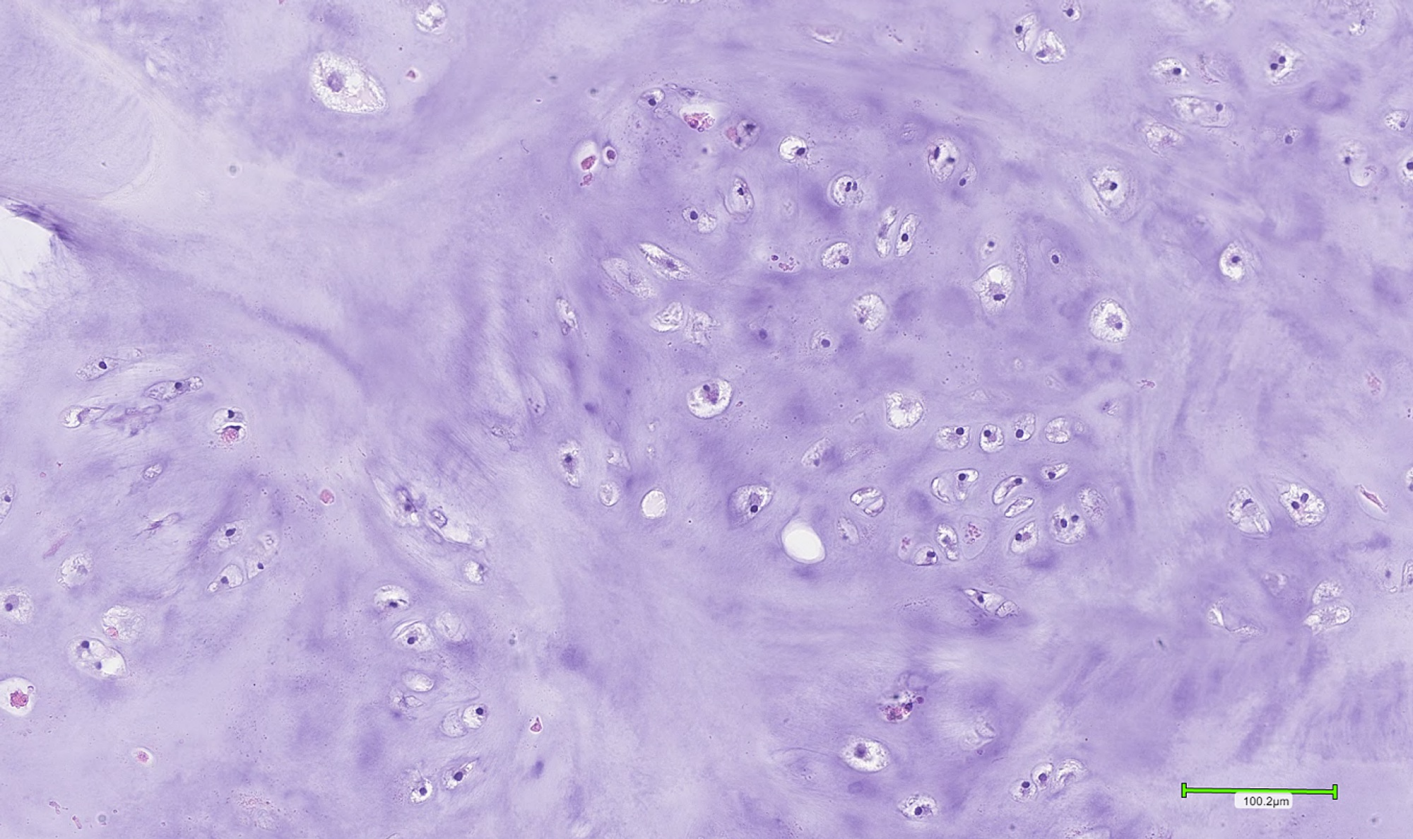
Section demonstrating groups of chondrocytes, with some binucleation visible.

**Figure 3:**
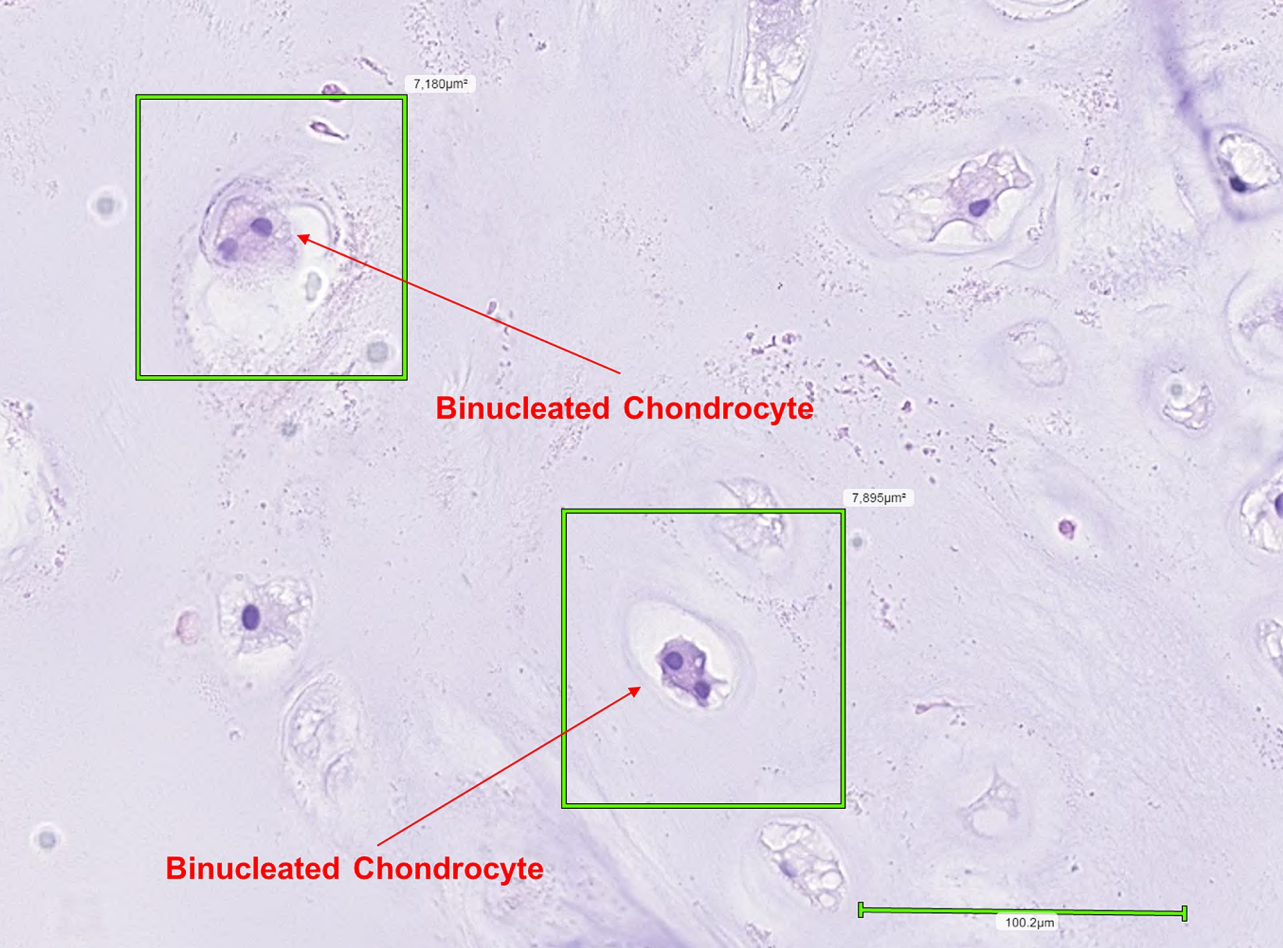
Section with two visible chondrocytes with binucleation.

**Figure 4:**
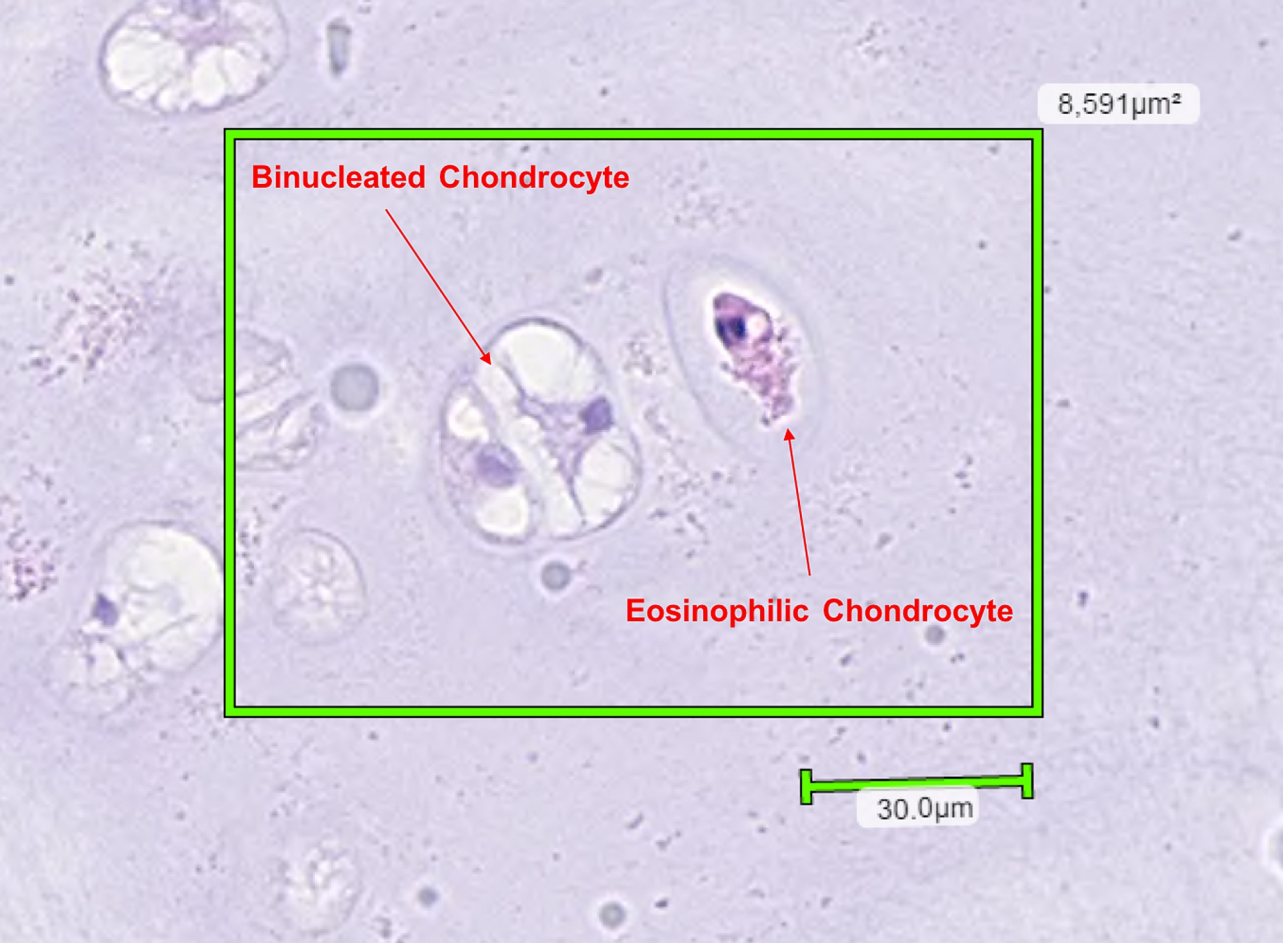
High powered view of a binucleated chondrocyte.

A ragged polypoid firm-tan white lesion resembling a cartilaginous tumour was observed upon gross examination. Histopathological examination revealed a well-circumscribed tumour composed of lobulated mature hyaline cartilage with slightly increased cellularity. The chondroid matrix was evenly distributed in a well-defined lobular pattern invested by fibrous septae (Figure 1). The majority of chondrocytes were arranged in individual lacunae and appeared bland with single, uniform, small nuclei surrounded by clear to eosinophilic cytoplasm (Figure 2). Binucleated chondrocytes were also identified (Figures 3-4). No signs of ischemic change, significant pleomorphism, mitoses, or necrosis were present. This was consistent with features of a benign chondroid neoplasm rather than a low-grade chondrosarcoma.

## Discussion

Chondromas of the nasal septum are a rare occurrence (Poster: Peer MS, 2019). Of those rare cases, 60% occur in patients under 50 years old, with the most common age group being early adulthood [1,4].

Nasal chondromas are often defined by their firm smooth texture, lobulated appearance, and well-defined boundaries [3,5]. As a result of these features, they can be misdiagnosed as a nasal polyp. Microscopically they are defined by small cartilage cells, pale vacuolated cytoplasm, small dark stained nuclei, and binucleate cartilage [1]. Nasal chondromas can also present with the erosion of surrounding cortical bone [5]. Associated symptoms can include nasal obstruction and epistaxis [4]. Despite their indolent nature, if allowed to grow large enough they can cause pressure effects on the maxilla and orbits [1].

Malignant tumours are twice as common as benign cartilaginous tumours [4]. Therefore, careful consideration must be given to differentiate a chondroma from a chondrosarcoma. Malignant features such as necrosis, pleomorphism, or mitoses should be ruled out. In particular, elements such as anisocytosis should be identified as they could signify a transition stage from a chondroma to a low-grade chondrosarcoma [3].

A number of theories exist regarding the origin of a chondroma. This includes the cell rest theory which states that chondromas originate from embryonic cartilage that was not resorbed during endochondral ossification [4]. Another theory, the traumatic theory, attributes the pathogenesis to past trauma and healing processes [3]. Although the traumatic theory is refuted by many, the reported case in this article presents a patient with a deviated septum due to untreated nasal fractures. Therefore, an association between trauma and the development of a nasal chondroma should be considered.

Diagnosis of a nasal chondroma involves a combination of imaging and histopathological examination. CT scans are used to assess growth in the circumstance that a chondroma will appear well-circumscribed and homogenous [4]. Multi-site biopsies are recommended as chondosarcomas may only be seen focally.

Surgical excision is the treatment of choice and different approaches may be utilized depending on the aspect of the nasal septum that is affected [1]. Cartilaginous benign tumours are radio-resistant, but radiotherapy may be considered in the case of malignancy. In rare cases (5%), chondromas can transform into malignant neoplasms [4]. A recurrence rate of 10%-15% is expected after excision, therefore long-term follow-up is recommended [5].

## Conclusions

Although rare, a chondroma of the nasal septum should be considered in the differential diagnosis of a nasal septum lesion, especially with a history of nasal trauma. Macroscopic and histopathological features differentiating a chondroma from a chondrosarcoma should be identified. The treatment of choice is surgical excision, but long-term follow-up is recommended.
